# Expression of *cry2Ah1* and two domain II mutants in transgenic tobacco confers high resistance to susceptible and Cry1Ac-resistant cotton bollworm

**DOI:** 10.1038/s41598-017-19064-5

**Published:** 2018-01-11

**Authors:** Shengyan Li, Zeyu Wang, Yiyao Zhou, Changhui Li, Guiping Wang, Hai Wang, Jie Zhang, Gemei Liang, Zhihong Lang

**Affiliations:** 1 Biotechnology Research Institute, Chinese Academy of Agricultural Sciences, Beijing, 100081 China; 2 State Key Laboratory for Biology of Plant Diseases and Insect Pests, Institute of Plant Protection, Chinese Academy of Agricultural Sciences, Beijing, 100193 China

## Abstract

To improve the novel *Bacillus thuringiensis* insecticidal gene *cry2Ah1* toxicity, two mutants *cry2Ah1-vp* (V354VP) and *cry2Ah1-sp* (V354SP) were performed. SWISS-MODEL analysis showed two mutants had a longer loop located between β-4 and β-5 of domain II, resulting in higher binding affinity with brush border membrane vesicles (BBMV) of *Helicoverpa armigera* comparing with Cry2Ah1. The *cry2Ah1*, *cry2Ah1-vp*, and *cry2Ah1-sp* were optimized codon usage according to plant codon bias, and named *mcry2Ah1*, *mcry2Ah1-vp*, and *mcry2Ah1-sp*. They were transformed into tobacco via *Agrobacterium*-mediated transformation and a total of 4, 8, and 24 transgenic tobacco plants were obtained, respectively. The molecular detection showed the exogenous gene was integrated into tobacco genome, and successfully expressed at the transcript and translation levels. Cry2Ah1 protein in transgenic tobacco plants varied from 4.41 to 40.28 μg g^−1^ fresh weight. Insect bioassays indicated that all transgenic tobacco plants were highly toxic to both susceptible and Cry1Ac-resistant cotton bollworm larvae, and the insect resistance efficiency to Cry1Ac-resistant cotton bollworm was highest in *mcry2Ah1-sp* transgenic tobacco plants. The results demonstrated that *cry2Ah1* was a useful Bt insecticidal gene to susceptible and Cry1Ac-resistant cotton bollworm and had potential application for insect biocontrol and as a candidate for pyramid strategy in Bt crops.

## Introduction

Genetically engineered crops expressing insecticidal proteins from the *Bacillus thuringiensis* (Bt) have been used commercially since 1996^1–3^. Bt crops have been proved that successfully apply plant genetic engineering technology to protect the economically important crops from infestation^4^. The wide use of transgenic Bt crops has reduced production costs and insecticide use, but analysis of more than a decade of global monitoring data reveals that the frequency of resistance alleles has increased substantially in some field population of *Helicoverpa zea*^5^, and some major pests which are resistant to Bt insecticidal proteins have been selected in the laboratory^6,7^. Thus the evolution of insects resistant to transgenic Bt crops presents a challenge.

Several deployment tactics design to delay resistance have been proposed. These strategies include the following: moderate toxin dosage to ensure survival of fraction of susceptible inserts, high toxin dosage to kill insects heterozygous for resistance, temporal or tissue-specific toxin expression, provision of nontoxic plants (refuge strategy) and combination/stacking/pyramid of toxins (pyramid strategy)^2^. The “refuge” and “pyramid” strategies are widely used among these strategies. To compare with “pyramid” strategy, “refuge” strategy is affected by more factors. At the present stage, “pyramid” strategy appears to be the best way to delay resistance^2^, but the stacking toxins should have different binding sites at pest midgut membrane to reduce the likelihood of cross-resistance^8–10^. Research indicated that the transgenic cotton Bollgard II (Monsanto 15985) expressing two Bt proteins (Cry1Ac+Cry2Ab) is found to be more toxic to lepidopterous pests than Bollgard (DP50B) expressing only one Bt protein (Cry1Ac)^11^. One of the most popular pyramided Bt traits in the commercial application is Genuity$R$ SmartStax^TM^ which expresses six Bt proteins: Cry1A.105/Cry2Ab2 (MON 89034), Cry1F (TC1507), Cry3Bb1 (MON 88017), Cry34/35Ab1 (DAS-59122)^12^.

So far, there are a large number of Cry genes to be cloned, but only a few of them are used commercially in Bt crops^13^. Thus it is important to explore new highly toxic Bt genes for future Bt crop development. *cry2Ah1* is a novel *Bacillus thuringiensis* insecticidal gene that was obtained by a pooled clone method from soil samples^14^. Previous study indicated that the Cry2Ah1 protein purified from *Escherichia coli* had a weight loss activity against *Ostrinia furnacalis*, and a growth inhibitory activity to both susceptible and Cry1Ac-resistant *Helicoverpa armigera* populations^14^. The Cry2Ah1 protein had a growth inhibitory activity to Cry1Ac-resistant cotton bollworm which implied the binding site of Cry2Ah1 might be different to the Cry1Ac in the cotton bollworm. Cry2Ah1-vp (V354VP) and Cry2Ah1-sp (V354SP) were mutanted from Cry2Ah1 by insertion of Proline behind Valine^354^ and replacement of Valine^354^ by Serine and Proline, respectively. Thus, they had the same carbon skeleton with Cry2Ab in mutation location. The Cry2Ah1 was 93.88% sequence identity with Cry2Ab which had been widely used commercially in Bt crops^15^. Similar to Cry2Ab, Cry2Ah1 also had a growth inhibitory activity to some key lepidopteran pests, such as *H. armigera* and *O. furnacalis*^14^. The *cry2Ah1* gene and mutated *cry2Ah1* genes have been patented.

In this study, we transformed codon-optimized *cry2Ah1*, *cry2Ah1-vp*, and *cry2Ah1-sp* genes into tobacco plants, respectively. Analysis showed that over-expression of *mcry2Ah1*, *mcry2Ah1-vp*, and *mcry2Ah1-sp* genes conferred transgenic tobacco plants with a high resistance to both susceptible and Cry1Ac-resistant cotton bollworm and the insect resistance efficiency to Cry1Ac-resistant cotton bollworm was highest in *mcry2Ah1-sp* transgenic tobacco plants. This suggested that *cry2Ah1* gene and mutants, especially *cry2Ah1-sp*, could potentially be candidate gene to develop novel stacking traits Bt crops.

## Results

### Toxicity and binding affinity of Cry2Ah1 protein and mutants with BBMV

**Figure 1:**
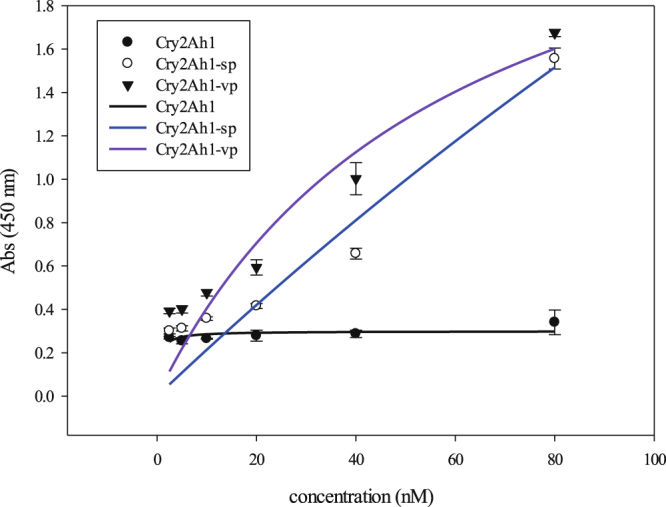
ELISA analyzed binding affinity of Cry2Ah1, Cry2Ah1-vp, and Cry2Ah1-sp proteins with *H. armigera* BBMV. *H. armigera* BBMV (1 μg) was used and incubated with increasing concentrations of toxins (0 to 80 nM). Data are means ± SEs (n = 3).

In previous studies^14,16^, the effective concentration 50 (EC50) of the Cry2Ah1 protein purified from *E. coli* was 8.74 μg mL^−1^ to *O. furnacalis*, 8.70 μg mL^−1^ to susceptible *H. armigera*, and 15.30 μg mL^−1^ to Cry1Ac-resistant *H. armigera*, respectively. The EC50 of Cry2Ah1-vp and Cry2Ah1-sp was 5.68 μg mL^−1^ and 1.63 μg mL^−1^ to susceptible *H. armigera*, respectively (Table 1). These results demonstrated Cry2Ah1-sp protein had the highest toxicity to susceptible *H. armigera* among three proteins. Furthermore, the binding of Cry2Ah1, Cry2Ah1-vp or Cry2Ah1-sp proteins with BBMV from *H. armigera* was measured by enzyme-linked immunosorbent assay (ELISA). The binding curve of Cry2Ah1 showed that there was no binding with BBMV, while Cry2Ah1-vp and Cry2Ah1-sp showed significant binding with BBMV but they were unsaturable (Fig. 1). This results suggested that Cry2Ah1 did not interact with BBMV, but its two mutants increased binding affinity with BBMV partially by insertion of Proline behind Valine^354^ and replacement of Valine^354^ by Serine and Proline.

**Table 1:** Toxicity of Cry2Ah1 and its mutants^14,16^.

Insect species	EC50 (FL95) (μg mL^−1^)		
	Cry2Ah1	Cry2Ah1-vp	Cry2Ah1-sp
*H. armigera* (S)	8.70 (7.56–9.87)	5.68 (3.91–8.50)	1.63 (0.64–3.01)
*H. armigera* (R)	15.30 (8.88–24.85)		
*O. furnacalis*	8.74 (6.81–11.02)

### SWISS-MODEL analysis of Cry2Ah1 and mutants three-dimensional structure

**Figure 2:**
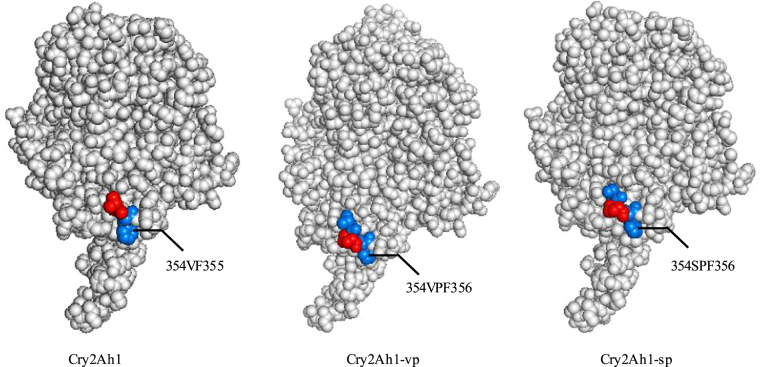
Simulation of three-dimensional structure of Cry2Ah1, Cry2Ah1-vp, and Cry2Ah1-sp proteins constructed by SWISS-MODEL (https://swissmodel.expasy.org). The homology gene Cry2Aa sequence was used as template for modeling. The images are licensed under the CC BY-SA 4.0 Creative Commons Attribution-ShareAlike 4.0 International License (https://creativecommons.org/licenses/by-sa/4.0/legalcode) and no change was made.

To localize the mutation region of Cry2Ah1-vp or Cry2Ah1-sp in structure, SWISS-MODEL (https://swissmodel.expasy.org) was employed to construct three-dimension structure by using the crystal structure of Cry2Aa (Fig. 2). The structures showed that Cry2Ah1-vp and Cry2Ah1-sp mutants had a longer loop located between β-4 and β-5 of domain II, which might contribute the higher growth inhibitory activity to *H. armigera* comparing with Cry2Ah1. These data suggested loop region between β-4 and β-5 of domain II was important but not sufficient for Cry2Ah1 binding with BBMV.

### Acquisition and identification of transgenic tobacco plants

**Figure 3:**
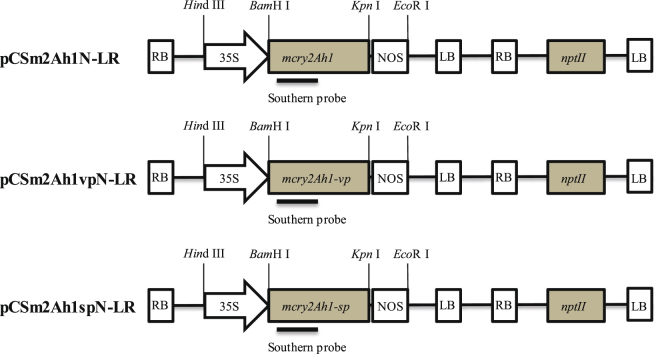
Schematic diagram of the transformation vectors. RB: right border of the T-DNA, 35 S: cauliflower mosaic virus 35 S promoter, *mcry2Ah1*, *mcry2Ah1-vp*, *mcry2Ah1-sp* represent modified *cry2Ah1* alleles, NOS: nopaline synthase gene terminator, LB: left border of the T-DNA, *nptII*: neomycin phosphotransferase II, a 862-bp fragment of the *mcry2Ah1* gene used as probe in Southern blot analysis.

**Figure 4:**
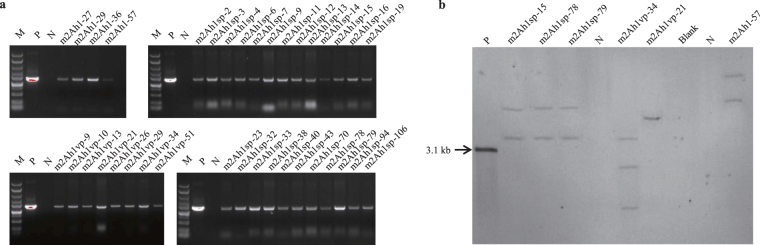
PCR and Southern blot analysis of the modified *cry2Ah1* alleles in transgenic tobacco plants. (**a**) PCR analysis of the *mcry2Ah1*, *mcry2Ah1-vp*, and *mcry2Ah1-sp* genes in corresponding transgenic tobacco plants. M: DNA marker, P: positive control, N: wild-type tobacco plants, (**b**) Southern blot analysis of six transgenic and wild-type tobacco plants. P: positive control, N: wild-type tobacco plants. The original full-length pictures were shown in Supplementary Figure S1.

*Agrobacterium tumefaciens* EHA105 carrying pCSm2Ah1N-LR, pCSm2Ah1vpN-LR, pCSm2Ah1spN-LR were used for transformation of tobacco NC89 leaf disks (Fig. 3). A total of 29, 45, and 62 regenerated tobacco plants were obtained, respectively. Out of these, 15, 28, and 46 regenerated tobacco plants were found to carry the *mcry2Ah1*, *mcry2Ah1-vp*, and *mcry2Ah1-sp* gene as can be detected by PCR assays (Table 2 and Fig. 4a). Genomic DNA of wild-type and transgenic tobacco plants was digested with *Hin*d III, for which there were no recognition sites in the probe region (Fig. 3). The Southern hybridization result showed that the *mcry2Ah1* genes were integrated into different sites of tobacco genome as a single copy (Fig. 4b, m2Ah1vp-21) or multiple copies (Fig. 4b, m2Ah1sp-15, 78,79, m2Ah1vp-34, m2Ah1–57) in transgenic tobacco plants and hybridization signal was not detected in the wild-type plants (Fig. 4b, N). PCR and southern hybridization results confirmed that the exogenous genes were transformed and integrated into the tobacco genome.

**Table 2:** Genetic transformation of tobacco with different plant expression vectors.

**Vector**	**No. of regenerated plants**	**No. of PCR-positive lines**	**No. of Cry2Ah1 expression lines**
pCSm2Ah1N-LR	29	15	4
pCSm2Ah1vpN-LR	45	28	8
pCSm2Ah1spN-LR	62	46	24

### Quantitation of Cry2Ah1 and mutants proteins in transgenic tobacco plants

**Figure 5:**
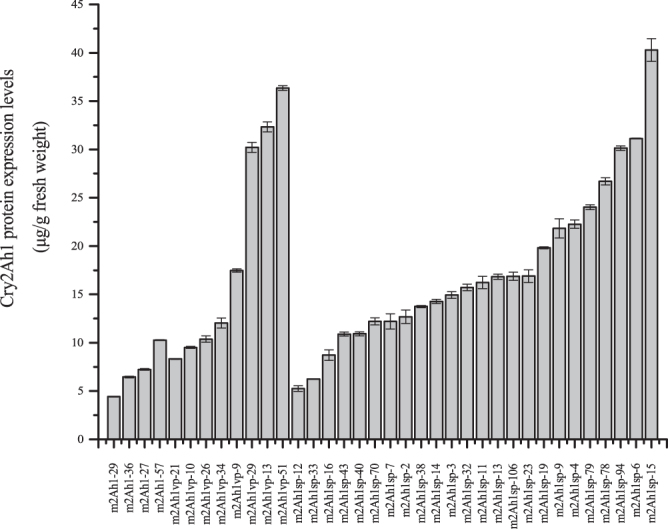
Enzyme-linked immunosorbent assay of Cry2Ah1 expression in different transgenic tobacco plants. m2Ah1, m2Ah1-vp, and m2Ah1-sp represent *mcry2Ah1*, *mcry2Ah1-vp*, and *mcry2Ah1-sp* plants, respectively. Data are means ± SEs (n = 3).

ELISA was used to quantitative detection of Cry2Ah1 protein expression in the above-mentioned 89 PCR-positive transgenic tobacco plants. Each treatment sample was carried on three technical replicates. Quantitative analysis revealed that 4 of *mcry2Ah1*, 8 of *mcry2Ah1-vp,* and 24 of *mcry2Ah1-sp* transgenic tobacco plants had a high Cry2Ah1 protein expression levels and the quantity of Cry2Ah1 endotoxin varied from 4.41 to 40.28 μg g^−1^ fresh weight (Table 3 and Fig. 5). The statistical analysis indicated that the Cry2Ah1 protein expression in *mcry2Ah1-sp* and *mcry2Ah1-vp* plants was not significantly different, but they were significantly higher than *mcry2Ah1* plants (Table 4).

**Table 3:** The corrected mortality rates of susceptible or Cry1Ac-resistant cotton bollworm larvae after 3 days of feeding on the leaves of transgenic tobacco plants.

Transgenic lines^a^	Cry2Ah1 expression^b^ (μg/g fresh weight)	Corrected mortality rates (%) of susceptible cotton bollworm^c^	Corrected mortality rates (%) of Cry1Ac-resistant cotton bollworm^c^
m2Ah1-27	7.23 ± 0.12	100.00 ± 0.00	95.42 ± 7.93
m2Ah1-29	4.41 ± 0.05	95.30 ± 8.14	90.84 ± 15.86
m2Ah1-36	6.45 ± 0.11	100.00 ± 0.00	93.34 ± 11.54
m2Ah1-57	10.27 ± 0.05	100.00 ± 0.00	100.00 ± 0.00
m2Ah1vp-9	17.47 ± 0.24	100.00 ± 0.00	100.00 ± 0.00
m2Ah1vp-10	9.51 ± 0.15	100.00 ± 0.00	94.77 ± 9.07
m2Ah1vp-13	32.32 ± 0.73	100.00 ± 0.00	100.00 ± 0.00
m2Ah1vp-21	8.32 ± 0.05	100.00 ± 0.00	93.85 ± 5.38
m2Ah1vp-26	10.38 ± 0.46	100.00 ± 0.00	92.09 ± 7.10
m2Ah1vp-29	30.21 ± 0.73	100.00 ± 0.00	100.00 ± 0.00
m2Ah1vp-34	12.04 ± 0.74	100.00 ± 0.00	93.62 ± 5.54
m2Ah1vp-51	36.35 ± 0.35	100.00 ± 0.00	100.00 ± 0.00
m2Ah1sp-2	12.69 ± 1.01	100.00 ± 0.00	100.00 ± 0.00
m2Ah1sp-3	14.93 ± 0.49	100.00 ± 0.00	96.95 ± 5.29
m2Ah1sp-4	22.27 ± 0.60	100.00 ± 0.00	100.00 ± 0.00
m2Ah1sp-6	31.13 ± 0.04	100.00 ± 0.00	100.00 ± 0.00
m2Ah1sp-7	12.22 ± 1.12	100.00 ± 0.00	100.00 ± 0.00
m2Ah1sp-9	21.83 ± 1.41	100.00 ± 0.00	100.00 ± 0.00
m2Ah1sp-11	16.23 ± 0.90	100.00 ± 0.00	97.38 ± 4.53
m2Ah1sp-12	5.26 ± 0.43	100.00 ± 0.00	96.34 ± 6.35
m2Ah1sp-13	16.82 ± 0.38	100.00 ± 0.00	97.18 ± 4.88
m2Ah1sp-14	14.27 ± 0.33	100.00 ± 0.00	96.34 ± 6.35
m2Ah1sp-15	40.28 ± 1.68	100.00 ± 0.00	100.00 ± 0.00
m2Ah1sp-16	8.72 ± 0.77	100.00 ± 0.00	96.34 ± 6.35
m2Ah1sp-19	19.82 ± 0.14	100.00 ± 0.00	96.95 ± 5.29
m2Ah1sp-23	16.89 ± 0.92	100.00 ± 0.00	96.95 ± 5.29
m2Ah1sp-32	15.72 ± 0.49	100.00 ± 0.00	100.00 ± 0.00
m2Ah1sp-33	6.23 ± 0.03	100.00 ± 0.00	97.96 ± 3.53
m2Ah1sp-38	13.75 ± 0.16	100.00 ± 0.00	96.34 ± 6.35
m2Ah1sp-40	10.92 ± 0.29	100.00 ± 0.00	96.34 ± 6.35
m2Ah1sp-43	10.89 ± 0.30	100.00 ± 0.00	96.34 ± 6.35
m2Ah1sp-70	12.21 ± 0.51	100.00 ± 0.00	96.67 ± 5.77
m2Ah1sp-78	26.70 ± 0.53	100.00 ± 0.00	100.00 ± 0.00
m2Ah1sp-79	24.03 ± 0.36	100.00 ± 0.00	100.00 ± 0.00
m2Ah1sp-94	30.13 ± 0.32	100.00 ± 0.00	100.00 ± 0.00
m2Ah1sp-106	16.87 ± 0.60	100.00 ± 0.00	100.00 ± 0.00

Cry2Ah1 expression levels are also indicated.

^a^m2Ah1, m2Ah1vp, and m2Ah1sp represent *mcry2Ah1*, *mcry2Ah1-vp*, and *mcry2Ah1-sp* transgenic tobacco plants, respectively.

^b^Cry2Ah1 protein expression levels of transgenic tobacco plants. Mean ± standard deviation values of three technical replicates from each line are shown.

^c^The corrected proportion of dead larvae to total larvae applied (%) of feeding on the leaves of transgenic tobacco plants. Mean ± standard deviation values of three biological replicates from each line are shown.

**Table 4:** Statistical analysis of the Cry2Ah1 expression levels and insect corrected mortality rates in transgenic tobacco lines.

Transgenic lines	Cry2Ah1 expression (μg/g fresh weight)	Corrected mortality rates (%) of susceptible cotton bollworm	Corrected mortality rates (%) of Cry1Ac-resistant cotton bollworm
m2Ah1	7.09 ± 2.43 a	98.83 ± 2.35 a	94.90 ± 3.88 a
m2Ah1vp	19.58 ± 11.53 b	100.00 ± 0.00 a	96.79 ± 3.51 a b
m2Ah1sp	17.53 ± 8.33 b	100.00 ± 0.00 a	98.25 ± 1.68 b

In the same column, the means followed by the same letters were not significantly different while different letters were significantly different (α = 0.05) according to the One-way ANOVA (least significant difference, LSD) using the SAS System (Version 8.1; SAS Institute Inc, Cary, North Carolina, USA).

### Relative quantitation of *mcry2Ah1* transcript levels

**Figure 6:**
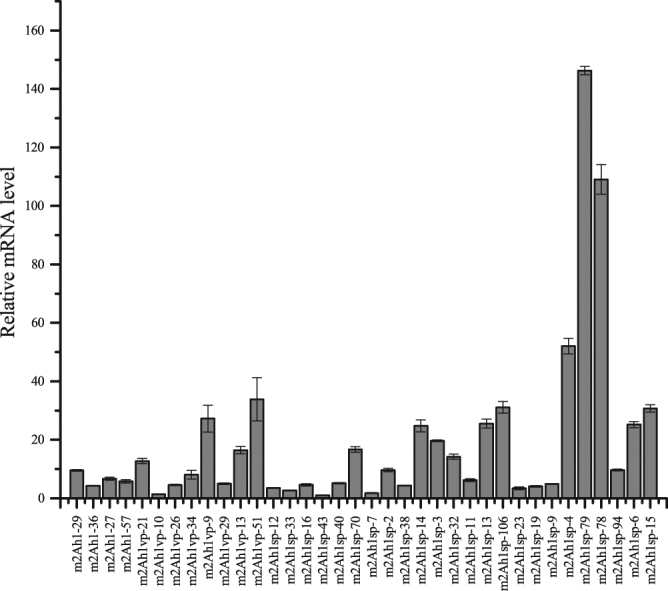
Quantitative RT-PCR analysis of *mcry2Ah1* transcript levels in transgenic tobacco plants. m2Ah1, m2Ah1-vp, and m2Ah1-sp represent *mcry2Ah1*, *mcry2Ah1-vp*, and *mcry2Ah1-sp* plants, respectively. The error bars represent the standard errors of three biological replicates from each transgenic line.

The *mcry2Ah1* transcript levels of 36 transgenic lines which had a high Cry2Ah1 protein expression were confirmed by quantitative RT-PCR. The tobacco *actin1* gene was used as a reference gene; the m2Ah1sp-43 transgenic tobacco plant was used as a calibrator. Each sample was carried on three technical replicates. The data were analyzed by 2^−△△Ct^ method. Quantitative RT-PCR results showed that the *mcry2Ah1* alleles were successfully transcripted in transgenic tobacco plants and the transcript levels were varied among the different transgenic lines (Fig. 6).

### Bioassay of transgenic tobacco plants harboring *cry2Ah1* gene and mutants

**Figure 7:**
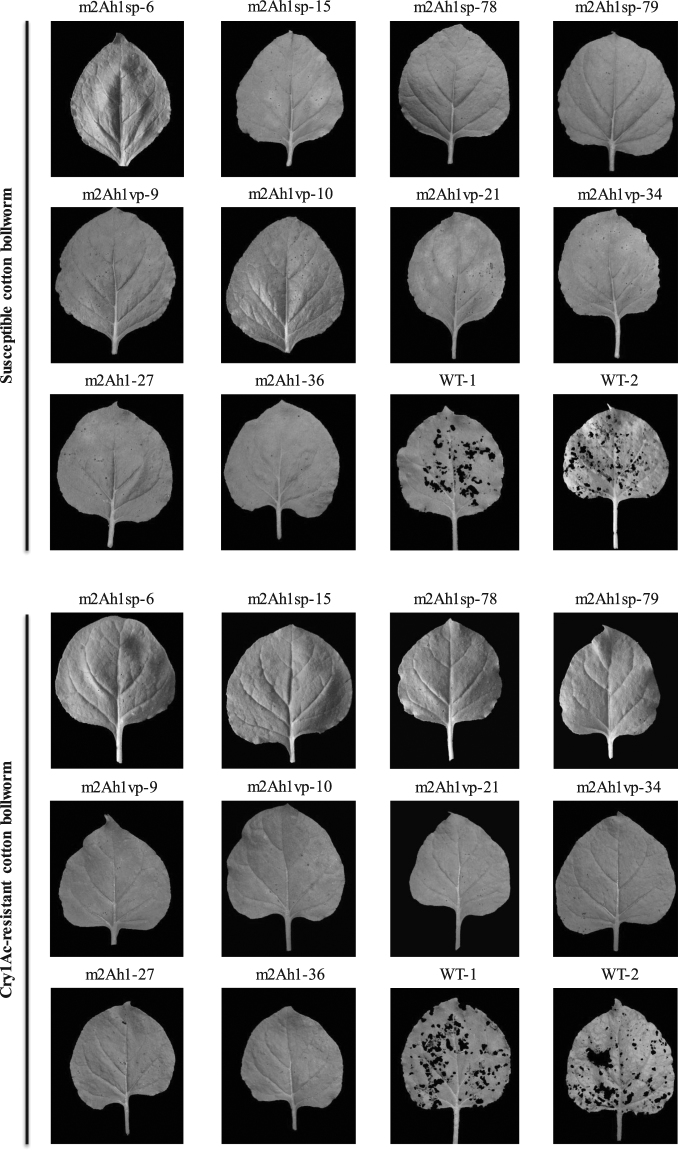
The appearance of control and transgenic tobacco leaves after insect bioassays with susceptible and Cry1Ac-resistant cotton bollworm. Leaves WT-1 and WT-2 were from wild-type tobacco plants, leaves m2Ah1-27 and m2Ah1-36 were from *mcry2Ah1* plants, leaves m2Ah1vp-9, m2Ah1vp-10, m2Ah1vp-21, and m2Ah1vp-34 were from *mcry2Ah1-vp* plants, leaves m2Ah1sp-6, m2Ah1sp-15, m2Ah1sp-78, and m2Ah1sp-79 were from *mcry2Ah1-sp* plants. Photographs were taken after 3 days of infestation.

To study whether transgenic tobacco producing Cry2Ah1 toxin could show the toxicity to the susceptible or Cry1Ac-resistant cotton bollworm, a total of 36 transgenic lines which had a high Cry2Ah1 protein expression were assessed along with two wild-type tobacco plants for control. The numbers of the living and dead cotton bollworm larvae were recorded every day and photographed the leaf damage at the end. The corrected mortality of cotton bollworm larvae was analyzed to assess the effect of the Cry2Ah1 protein on the larvae. After three days of infestation with the neonatal larvae, the transgenic tobacco plants showed significant resistance to both susceptible and Cry1Ac-resistant cotton bollworm compared to wild-type plants (Fig. 7). The wild-type leaves were badly damaged and most larvae were survived (Fig. 7, WT-1, 2). On the contrary, all transgenic plants leaves exhibited slightly damage and the vast majority of larvae were died and the growth of surviving larvae was significantly inhibited (Fig. 7). The corrected mortality rates of susceptible cotton bollworm larvae feeding on *mcry2Ah1*, *mcry2Ah1-vp*, and *mcry2Ah1-sp* plants were 95.30–100%, 100%, and 100%, respectively (Table 3). The statistical analysis showed that the corrected mortality rates of susceptible cotton bollworm larvae feeding on *mcry2Ah1*, *mcry2Ah1-vp*, and *mcry2Ah1-sp* plants were not significantly different (Table 4). To Cry1Ac-resistant cotton bollworm, the corrected mortality rates of larvae feeding on *mcry2Ah1*, *mcry2Ah1-vp*, and *mcry2Ah1-sp* plants after 3 days of infestation were 90.84–100%, 92.09–100% and 96.34–100%, respectively (Table 3). The statistical analysis showed that the corrected mortality rates of Cry1Ac-resistant cotton bollworm larvae feeding on *mcry2Ah1* and *mcry2Ah1-sp* plants were significantly different; the *mcry2Ah1-sp* plants had a higher toxic than *mcry2Ah1* plants. The corrected mortality rates of Cry1Ac-resistant cotton bollworm larvae were no significant difference between *mcry2Ah1-vp* and *mcry2Ah1*, *mcry2Ah1-vp* and *mcry2Ah1-sp*, but the corrected mortality rates feeding on *mcry2Ah1-vp* plants were higher than *mcry2Ah1*, lower than *mcry2Ah1-sp*. (Table 4). The results of the insect bioassays indicated that all transgenic tobacco plants were toxic to both susceptible and Cry1Ac-resistant cotton bollworm, the insect resistance efficiencies to susceptible cotton bollworm were not different among *mcry2Ah1*, *mcry2Ah1-vp*, and *mcry2Ah1-sp* plants, the insect resistance efficiency to Cry1Ac-resistant cotton bollworm was highest in *mcry2Ah1-sp* plants followed by *mcry2Ah1-vp* and *mcry2Ah1* plants, respectively.

## Discussion

The *cry2Ah1* is a novel *Bacillus thuringiensis* insecticidal gene that was obtained by a pooled clone method from soil samples^14^. It encoded a polypeptide of 632 amino acids with three domains which had 18.50%, 19.62%, and 93.88% sequence identity with Cry1Ab (AAA22561.1), Cry1Ac (AAA22331.1), and Cry2Ab (ABC95996.1), respectively. The previous research show that Cry2Ah1 protein purified from *E. coli* has a growth inhibitory activity to lepidopteran pests, including *O. furnacalis* and *H. armigera*^14^. Two mutants of Cry2Ah1, Cry2Ah1-vp and Cry2Ah1-sp, also have a growth inhibitory activity to *H. armigera*^16^.

In order to verify the insecticidal activity of Cry2Ah1 in plants, we modified the *cry2Ah1* gene according to the plant codon bias (named *mcry2Ah1*) and created its two mutants (*mcry2Ah1-vp*, *mcry2Ah1-sp*) by overlap-PCR. We generated transgenic tobacco lines expressing the *mcry2Ah1*, *mcry2Ah1-vp*, and *mcry2Ah1-sp* alleles via *Agrobacterium*-mediated transformation. The results of molecular detection showed that the *cry2Ah1* gene was inserted into the genome of tobacco and could be expressed normally (Figs 4, 5 and 6). In our study, we found the transcript and translation levels did not show a similar trend in transgenic tobacco plants. The correlation of mRNA-protein is not strictly linear due to the synthesis and degradation rates may affect the amount of mRNA and protein differentially^17^. Insect bioassays showed the transgenic tobacco plants harboring the *mcry2Ah1*, *mcry2Ah1-vp*, and *mcry2Ah1-sp* were highly toxic to the susceptible cotton bollworm larvae, the corrected mortality rates were not significantly different among them (Table 4). The results indicated that the *cry2Ah1* was a highly toxic Bt gene, it could be used for the development of Bt crops. In addition, unlike purified from *E. coli*, the Cry2Ah1 protein expressing in tobacco plants had a high insecticidal activity to cotton bollworm. It was possible that the Cry2Ah1 protein purified from *E. coli* did not have the right active form.

In the two decades leading up to the first commercial release of Bt plants, an unprecedented cumulative hectarage of 2 billon hectares are successfully cultivated globally^3^. In China, Bt cotton producing Cry1Ac has been commercialized for 20 years^18^, and now the adoption rate of transgenic Bt cotton is nearly 96%^3^. As a result of Bt genes were used commercially, the evolution of resistance by pests is the primary threat to the continued efficacy of Bt crops^5,19–22^. Many reports have shown that mutations in midgut receptors are related to Cry1A resistance in *Heliothis virescens*, *H. armigera*, and *Pectinophora gossypiella*^7,23^. Therefore, it is important to identify new Bt proteins for the sustainable use of Bt crops, which should have different modes of action. Although the EC50 of Cry2Ah1 is lower than Cry1Ac to *O. furnacalis* or *H. armigera*^24^, the previous research showed Cry2Ah1 protein purified from *E.coli* is toxic to Cry1Ac-resistant cotton bollworm^14^. In the study, the transgenic tobacco lines producing the Cry2Ah1, Cry2Ah1-vp, and Cry2Ah1-sp were highly toxic to Cry1Ac-resistant cotton bollworm and Cry2Ah1-sp had the highest insecticidal activity. It confirmed that Cry2Ah1 did not compete for the Cry1Ac binding site in the cotton bollworm. The Cry1Ac-resistant population of *H. armigera* was provided by Wu’s lab. The mechanisms of resistance to Cry1Ac were some receptors’ mutation, such as mutation disrupting an ATP-binding cassette protein (ABCC2), mutations disrupting the domains of cadherin, mutation reducing aminopeptidase N (APN) activity, and unidentified genes^25–28^. This suggested that *cry2Ah1*, especially *cry2Ah1-sp*, might be a good candidate for development of stack trait Bt crops.

The insect resistance efficiency to Cry1Ac-resistant cotton bollworm was highest in *mcry2Ah1-sp* transgenic tobacco plants (Table 4), indicated that the Cry2Ah1-sp protein had the highest toxicity among Cry2Ah1, Cry2Ah1-vp, and Cry2Ah1-sp. The differences were 354th amino acid located in loop between β-4 and β-5 of domain II among Cry2Ah1, Cry2Ah1-vp, and Cry2Ah1-sp. The loops of Cry protein domain II are always involved in the specificity of toxin binding to the receptor^29,30^. Mutations of amino acids in domain II may have either a negative or positive effect on binding and toxicity^31^. Substitution of domain II residue N372 with Ala or Gly (N372A, N372G) increase the toxicity of Cry1Ab against gypsy moth larvae 8-fold and enhance binding affinity to gypsy moth midgut BBMV about 4-fold^32^. In this study, we found Cry2Ah1 was no binding affinity with BBMV of *H. armigera*, while Cry2Ah1-vp (V354VP) and Cry2Ah1-sp (V354SP) showed significant binding affinity with BBMV (Fig. 1), resulting in significantly increased the toxicity of Cry2Ah1-vp and cry2Ah1-sp against Cry1Ac-resistant cotton bollworm larvae. This change made the mutants had a longer loop located between β-4 and β-5 of domain II and enhance the binding affinity to BBMV (Fig. 2).

Overall, this study clearly showed that *cry2Ah1* and mutants was a useful Bt insecticidal gene to lepidopteran larve. Its high level of toxicity to lepidopteran larve and no cross-resistance with Cry1Ac made it potentially useful for insect biocontrol and as a candidate for pyramid strategy in Bt crops.

## Methods

### Expression and purification of Cry2Ah1 protein and mutants

Expression of Cry2Ah1 protein and Cry2Ah1-vp and Cry2Ah1-sp in 300 mL LB medium of *E. coli* was induced with 0.5 mM isopropyl-β-D-thiogalactopyranoside (IPTG) at 16 °C overnight, and then cells were collected by centrifugation at 10,000 g for 8 min and the cell pellet was resuspended in 30 mL of binding buffer (20 mM Tris-HCl with 0.5 M NaCl and 50 mM imidazole, pH 8.0). Cells were then lysed by ultrasonication (Ningbo Scientz Biotechnology Co., Ltd., Ningbo, China) for 6 min (70% power, 3-s pulse on, 5-s pulse off) and then centrifuged at 13,000 g for 15 min at 4 °C. Cry2Ah1 toxin and Cry2Ah1-vp and Cry2Ah1-sp were solubilized from inclusion bodies with 5 mL urea buffer (8 M urea, 10 mM Tris-HCl and 100 mM NaH_2_PO_4_). These three proteins were purified by Ni-affinity chromatography (GE Healthcare, Uppsala, Sweden), then preequilibrated with urea buffer. Nonspecifically adsorbed proteins were removed by washing with decreased concentration urea buffer (6 M urea, 4 M urea and 2 M urea) orderly. Proteins bound to the column were eluted with elution buffer (20 mM Tris-HCl, 500 mM NaCl, 250 mM imidazole, pH 8.0).

### Preparation of brush border membrane vesicles (BBMV)

*H. armigera* (Hübner) midgut tissues from third instar larvaes were dissected and stored immediately at −70 °C. BBMV were prepared by the magnesium precipitation method as described by Wolfersberger *et al*.^33^ and stored at −70 °C until used. Purity of BBMV preparations was determined by estimating the enrichment of aminopeptidase N (APN) specific activity in the BBMV compared to that in initial midgut tissue homogenates as described by Lorence *et al*.^34^. Representative APN activity enrichment in the final BBMV preparations was 3- to 4-fold compared to initial midgut homogenates.

### ELISA analysis of Cry2Ah1 protein and mutants with BBMV binding

In binding assay, ELISA plates, 96 wells, were incubated 12 h at 4 °C with 1 µg BBMV in PBS (pH 7.4), followed by 5 times wash with PBS. The plates were then incubated with PBST with 2% BSA for 2 h at 37 °C. The ELISA plates were incubated, subsequently, with different concentrations of Cry2Ah1, Cry2Ah1-vp or Cry2Ah1-sp proteins (0, 2.5, 5, 10, 20, 40 and 80 nM) for 1 h at 37 °C, after 5 times wash with PBST, then detected with anti-Cry2Ah antibody (1: 5,000 dilution) for 1 h at 37 °C. Goat-anti mouse secondary antibody was incubated with plate in dilution of 1: 20, 000 (Sigma-Aldrich, St. Louis, USA). The enzymatic activity was revealed with TMB Single-Component Substrate solusion (Solarbio, Beijing, China). The enzymatic reaction was stopped with 2 M HCl, and the absorbance was read at 450 nm. Binding data were analyzed and plotted with SigmaPlot v.12.5 software.

### SWISS-MODEL analysis of Cry2Ah1 and mutants three-dimensional structure

The three-dimensional structures of Cry2Ah1 and mutants were calculated with the SWISS-MODEL Server (Swiss Institute of Bioinformatics, Lausanne, Switzerland, https://swissmodel.expasy.org)^35,36^. The homology gene Cry2Aa sequence was used as template for modeling. The images are licensed under the CC BY-SA 4.0 Creative Commons Attribution-ShareAlike 4.0 International License (https://creativecommons.org/licenses/by-sa/4.0/legalcode) and no change was made.

### Codon-optimized of *cry2Ah1* gene and construction of transformation vectors

According to plant codon usage pattern, we optimized the codon of *cry2Ah1* (EU939453.1) gene. The variations of codon usage in the optimized *cry2Ah1* gene are shown in Table 5. The GC content was increased from the original 34% to 61.6%. The sequence of optimized *cry2Ah1* gene was synthesized by GenScript Corporation (Nanjing, China). The 1899 bp *mcry2Ah1* gene was digested by *Bam*H I (5′) and *Kpn* I (3′), and subsequently inserted into *Bam*H I/*Kpn* I-digested binary vector pCSN-LR which was modified from pCAMBIA2300, with a CaMV35S promoter and Nos terminator. The resulting binary vector is named as pCSm2Ah1N-LR and has the *nptII* as plant selection marker gene. The *mcry2Ah1-vp* gene and *mcry2Ah1-sp* gene were cloned by overlap-PCR and constructed into pCSN-LR according to the protocol of *mcry2Ah1*, named pCSm2Ah1vpN-LR and pCSm2Ah1spN-LR, respectively.

**Table 5:** Codon usage variations in optimized *cry2Ah1* gene.

Preference of codon	Original *cry2Ah1* (%)	Optimized *cry2Ah1* (%)
F (TTC)	11	100
L (CTC)	2	52
I (ATC)	13	100
V (GTG)	19	55
S (TCC)	6	51
T (ACC)	11	56
A (GCC)	9	25
Y (TAC)	15	96
H (CAC)	33	92
Q (CAG)	23	100
N (AAC)	12	100
K (AAG)	10	100
D (GAC)	8	100
E (GAG)	30	100
C (TGC)	0	100
R (AGG)	5	59
G (GGC)	12	100

### Tobacco materials and transformation

Wild-type tobacco (NC89) was selected as experimental materials. The tobacco’s growth and tissue culture according to the protocol described by Li *et al*.^37^. Three plant expression vectors pCSm2Ah1N-LR, pCSm2Ah1vpN-LR, and pCSm2Ah1spN-LR were transformed into tobacco via *Agrobacterium*-mediated transformation^38^. Kanamycin (100 mg L^−1^) was the selection agent on Murashige and Skoog (MS) regeneration medium^39^. PhytoTechnology Laboratories (Shawnee Mission, KS, USA) provided the plant tissue culture reagents.

### PCR and southern blot analysis

Genomic DNA was isolated from the leaf tissues by CTAB method^40^. The following PCR primers were used to detection of *mcry2Ah1*, *mcry2Ah1-vp*, and *mcry2Ah1-sp* genes: forward primer 5′-CCTCATCTTCCCGTC-3′, and reverse primer 5′-GTGTTGCTCTGCTCG-3′. The 20-μL reaction mixtures contained 100 ng of DNA template, 0.1 μM of each primer, 10 μL 2× Taq MasterMix (Dye) (CWBIO, Beijing, China). The PCR reaction conditions were as follows: 2 min at 94 °C, followed by 30 cycles of 30 s at 94 °C, 30 s at 56 °C and 60 s at 72 °C. A final extension step (10 min at 72 °C) was added to all reactions. The PCR products were visualized and documented on Gel Doc^TM^ XR+ system (Bio-Rad Laboratories, California, USA). More than 30 μg of genomic DNA of wild-type and each transgenic tobacco plant were used for the southern blot analysis. They were digested with *Hin*d III and electrophoresed on a 0.7% (w/v) agarose gel and blotted on Hybond^TM^-N^+^ nylon membranes (GE Healthcare, UK). A 862-bp fragment of the *mcry2Ah1* gene was used as probe in Southern blot analysis and it was amplified from pCSm2Ah1N-LR by PCR using the following primers: forward primer 5′- GAGTGGATGGAGTGGAAG-3′, and reverse primer 5′- CGATGTTTGGGAAGGTCT-3′. The probe DNA was labeled with DIG-11-dUTP using the PCR DIG Probe Synthesis Kit (Roche, Mannheim, Germany). The immunological detection was carried out as the protocol of DIG High Prime DNA labeling and Detection Starter Kit II (Roche, Mannheim, Germany). The image analysis was carried out in Tanon 5200 automatic chemiluminescence imaging analysis system (YPH-BIO, Beijing, China).

### ELISA analysis of Cry2Ah1 protein expression in transgenic tobacco plants

QuantiPlate^TM^ Kit for Cry2A (Envirologix, Portland, USA) was used for quantitative detection of Cry2Ah1 protein in fresh leaf tissues. 100 mg of tobacco leaf tissues were homogenized and used for detection of Cry2Ah1 protein as per the manufacturer’s protocol. The OD was measured at 450 nm using BioTek Elx808 (BioTek, Winooski, USA).

### Quantitative RT-PCR analysis

Total RNA was isolated from 100 mg of leaf tissues using Trizol reagents (TransGen, Beijing, China). Approximately 2 μg of total RNA was used as a template for reverse transcription using RevertAid First Strand cDNA Synthesis Kit (Thermo Scientific, Waltham, USA) according to the manufacturer’s instructions. Quantitative RT-PCR was performed using an Applied Biosystems^®^ QuantStudio^®^ 3 Real-Time PCR System (ThermoFisher Scientific, Waltham, USA). The tobacco *actin* (X63603.1) gene was used as a reference gene. All reactions were run as duplicates in 96-well plates. The 20-μL reaction mixtures contained 10 μL of 2× TransStart^®^ Top Green qPCR SuperMix (TransGen, Beijing, China), 0.2 μM of each primer and 1 μL of cDNA template. The reaction conditions were as follows: 30 s at 95 °C, 40 cycles of 5 s at 95 °C and 34 s at 60 °C, followed by a melting curve analysis. Primer pair mcry2Ah1-qF1/R1 was used to detect *mcry2Ah1*, *mcry2Ah1-vp*, and *mcry2Ah1-sp* transcript, respectively, and primer pair actin-qF1/R1 was used to detect the *actin1* transcript. The sequence of primers were mcry2Ah1-qF1: 5′-AGGGCGTACATGGTGAGC-3′, mcry2Ah1-qR1: 5′-GGATGGGGGAGATGGTGA-3′, actin-qF1: 5′-GGCATCATACATTTTACAACGAA-3′, actin-qR1: 5′-ATGGCGACATACATAGCAGGAGT-3′.

### Cotton bollworm bioassays

To determine the resistance of transgenic tobacco plants to susceptible and Cry1Ac-resistant cotton bollworm (*H. armigera)*. The neonatal larvae of susceptible and Cry1Ac-resistant cotton bollworm and the leaves of 1-month-old transgenic tobacco plants were used in the bioassay. The resistance level of Cry1Ac-resistant cotton bollworm reached 2917.33-fold^41^. Each fresh leaf was infested with 12 neonate insect larvae and kept in the rearing room at 25 °C, 16:8 light:dark period. The numbers of the living and dead cotton bollworm larvae were recorded every day. Each treatment was performed three biological replicates and wild-type tobacco plants (NC89) as a control. Mortality rates were presented as the proportion of dead larvae to total larvae applied (%). Corrected mortality rates were calculated in the equation (1).

1 $${\rm{Corrected}}\,{\rm{mortality}}\,{\rm{rates}}( \% )=\frac{({\rm{mortality}}\,{\rm{rates}}\,{\rm{of}}\,{\rm{treatment}}\,{\rm{group}})-({\rm{mortality}}\,{\rm{rates}}\,{\rm{of}}\,{\rm{control}}\,{\rm{group}})}{{\rm{100}}-({\rm{mortality}}\,{\rm{rates}}\,{\rm{of}}\,{\rm{control}}\,{\rm{group}})}\,\ast \,\mathrm{100} \% $$

## Electronic supplementary material

Supplementary information
