# Supplementary material for: Expression of cry2Ah1 and two domain II mutants in transgenic tobacco confers high resistance to susceptible and Cry1Ac-resistant cotton bollworm
Source: Sci Rep. 2018 Jan 11;8:508. doi: 10.1038/s41598-017-19064-5 (PMC5765002; doi:10.1038/s41598-017-19064-5)
Supplement: Supplementary file 1 — Supplementary information [file 41598_2017_19064_MOESM1_ESM.pdf]

Supplementary Information

Expression of *cry2Ah1* and two domain II mutants in transgenic tobacco confers high resistance to susceptible and Cry1Ac-resistant cotton bollworm

Shengyan Li[1†], Zeyu Wang[2†], Yiyao Zhou[1], Changhui Li[2], Guiping Wang[1], Hai Wang[1], Jie Zhang[2], Gemei Liang[2], Zhihong Lang[1*]

1 Biotechnology Research Institute, Chinese Academy of Agricultural Sciences, Beijing 100081, China
2 State Key Laboratory for Biology of Plant Diseases and Insect Pests, Institute of Plant Protection, Chinese Academy of Agricultural Sciences, Beijing 100193, China
† These authors contributed equally to this work.
* To whom correspondence should be addressed (email: langzhihong@caas.cn)

a

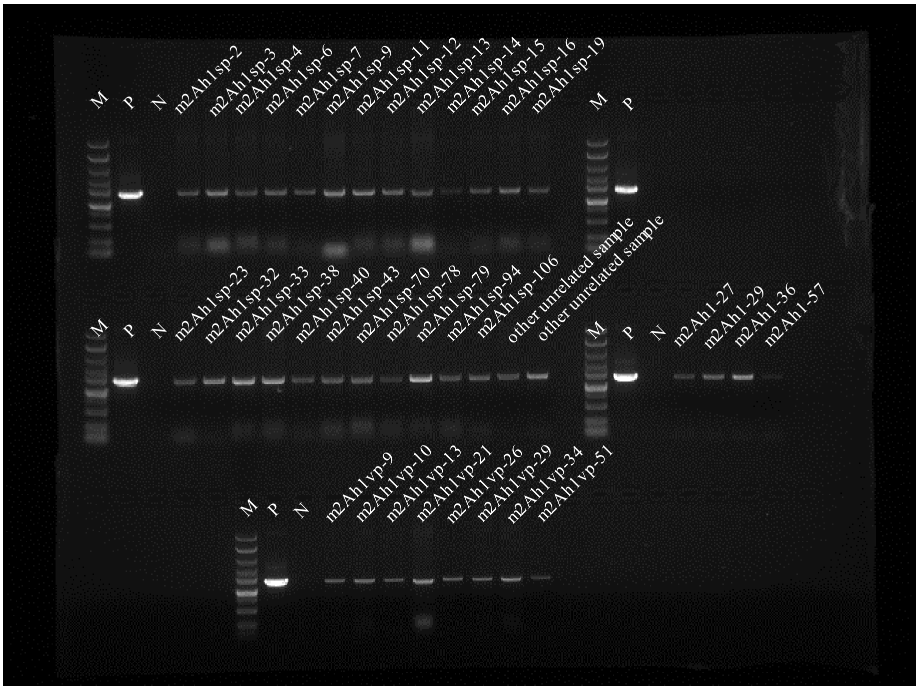

b

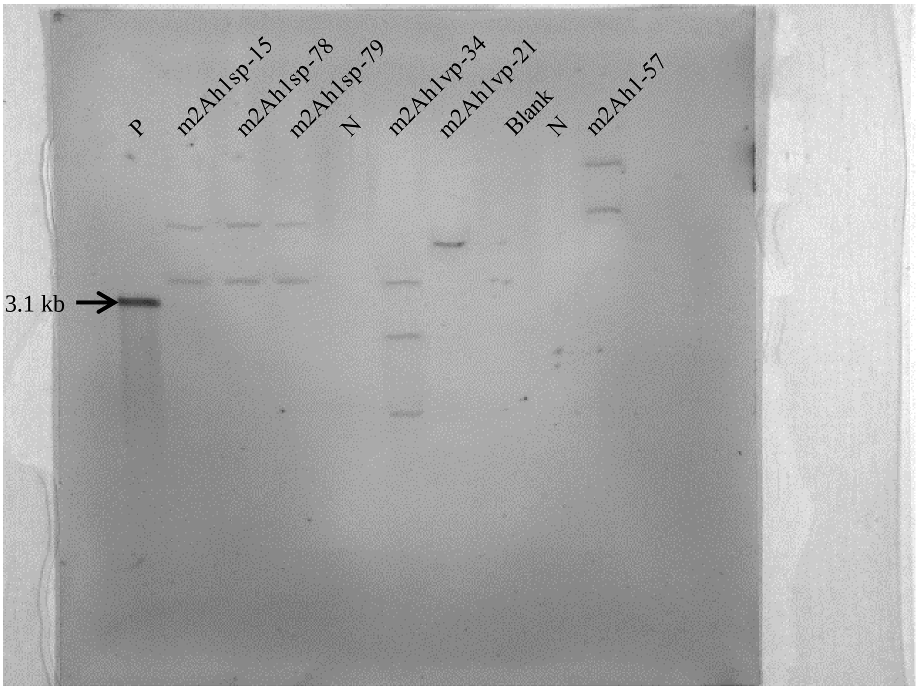

Figure S1 Original full-length pictures of PCR and Southern blot analysis. **a** PCR analysis of the *mcry2Ah1*, *mcry2Ah1-vp*, and *mcry2Ah1-sp* genes in corresponding transgenic tobacco plants. M: DNA marker, 0.1-10 kb (CWBIO, Beijing, China), P: positive control, N: wild-type tobacco plants, **b** Southern blot analysis of six transgenic and wild-type tobacco plants. P: positive control, N: wild-type tobacco plants.
